# Arabidopsis bHLH100 and bHLH101 Control Iron Homeostasis via a FIT-Independent Pathway

**DOI:** 10.1371/journal.pone.0044843

**Published:** 2012-09-11

**Authors:** Alicia B. Sivitz, Victor Hermand, Catherine Curie, Grégory Vert

**Affiliations:** 1 Biochimie et Physiologie Moléculaire des Plantes, Centre National de la Recherche Scientifique Unité Mixte de Recherche 5004, Institut de Biologie Intégrative des Plantes, Montpellier, France; 2 Department of Biological Sciences, Dartmouth College, Hanover, New Hampshire, United States of America; 3 Plant Biology Laboratory, The Salk Institute for Biological Studies, La Jolla, California, United States of America; University of Nottingham, United Kingdom

## Abstract

Iron deficiency induces a complex set of responses in plants, including developmental and physiological changes, to increase iron uptake from soil. In Arabidopsis, many transporters involved in the absorption and distribution of iron have been identified over the past decade. However, little is known about the signaling pathways and networks driving the various responses to low iron. Only the basic helix–loop–helix (bHLH) transcription factor FIT has been shown to control the expression of the root iron uptake machinery genes *FRO2* and *IRT1*. Here, we characterize the biological role of two other iron-regulated transcription factors, bHLH100 and bHLH101, in iron homeostasis. First direct transcriptional targets of FIT were determined *in vivo*. We show that bHLH100 and bHLH101 do not regulate FIT target genes, suggesting that they play a non-redundant role with the two closely related bHLH factors bHLH038 and bHLH039 that have been suggested to act in concert with FIT. bHLH100 and bHLH101 play a crucial role in iron-deficiency responses, as attested by their severe growth defects and iron homeostasis related phenotypes on low-iron media. To gain further insight into the biological role of bHLH100 and bHLH101, we performed microarray analysis using the corresponding double mutant and showed that bHLH100 and bHLH101 likely regulate genes involved in the distribution of iron within the plant. Altogether, this work establishes bHLH100 and bHLH101 as key regulators of iron-deficiency responses independent of the master regulator FIT and sheds light on new regulatory networks important for proper growth and development under low iron conditions.

## Introduction

Iron deficiency is a major issue worldwide for humans and plants. Since a majority of humans derive dietary iron from plant sources, it is vital to understand how plants sense, take up, distribute internally, and make use of iron for metabolic processes such as respiration and photosynthesis. Iron is often poorly available to plants in soils so plants have evolved mechanisms to respond to iron-deficient conditions. Non-grass species like the model plant *Arabidopsis thaliana* are known to respond to low-iron conditions by upregulating genes necessary to remobilize soil iron and transport it into root cells. Remobilization of soil iron is accomplished by activating i) the AHA2 proton pump that acidifies the rhizosphere to release iron from soil complexes [1], ii) the FRO2 ferric-iron reductase that reduces rhizosphere iron to the ferrous form, and [2] and iii) the IRT1 ferrous-iron uptake transporter that imports iron into root cells [3]. The genes required for this remobilization and uptake have been shown to be under the control of the bHLH transcription factor FIT [4].

Several studies have established FIT as a master regulator of iron-deficiency responses playing a crucial role in iron uptake in Arabidopsis. Loss-of-function mutations of *FIT* cause seedling lethality unless watered with copious amounts of iron [4]–[6]. The *FIT* gene is only expressed in roots and has been shown to be regulated by iron starvation both transcriptionally and post-translationally [4]–[8]. FIT has been proposed to autoregulate itself since the *FIT* promoter is less active in the *fit-3* background compared to wild-type plants [5], [9].

Other than FIT, only a few transcription factors involved in iron-deficiency responses have been identified. PYE, another bHLH transcription factor was recently identified and shown to control the expression of many genes involved in iron-deficiency responses that were not found to be regulated by FIT [10]. A group of four closely-related bHLH genes belonging to the clade Ib of the bHLH superfamily from Arabidopsis, bHLH038, bHLH039, bHLH100 and bHLH101, have been shown to be upregulated by low iron, but their function has remained elusive. Individual loss-of-function mutations in these genes showed no phenotype, which was attributed to their redundancy [9]. Unlike FIT, which is expressed solely in roots, the four bHLH genes were found to be broadly expressed in both roots and shoots [9]. Overexpressing FIT alone is not sufficient to trigger the expression of the root iron uptake genes FRO2 and IRT1 [11]. However, co-overexpression of FIT together with bHLH038 or bHLH039, that were shown to dimerize with FIT, leads to constitutive expression of FRO2 and IRT1 [11]. FIT was therefore proposed to work in concert with bHLH038 and bHLH039 to control root iron-deficiency responses. However, final proof using loss-of-function approaches to confirm the involvement of bHLH038 and bHLH039 in root iron uptake is still lacking. Although, bHLH100 and bHLH101 were suggested, by analogy with bHLH038 and bHLH039, to interact with FIT to control root genomic responses, no data is currently available to support such a model.

To shed light on the precise biological role of bHLH100 and bHLH101, we first identified FIT direct targets *in vivo* and showed that *bhlh100/bhlh101* loss-of-function had no influence on FIT-target gene expression, suggesting that bHLH100 and bHLH101 act independently of FIT. However, bHLH100 and bHLH101 play a crucial role in plant responses to iron starvation since the *bhlh100/bhlh101* double mutants display typical iron-deficiency hallmarks of chlorosis and growth defects. Genome-wide changes in gene expression associated with the loss of bHLH100 and bHLH101 indicated that both transcription factors control iron homeostasis likely by affecting the distribution of iron between tissues and organelles.

## Results

### Determination of FIT Direct Targets *in vivo*

**Figure 1:**
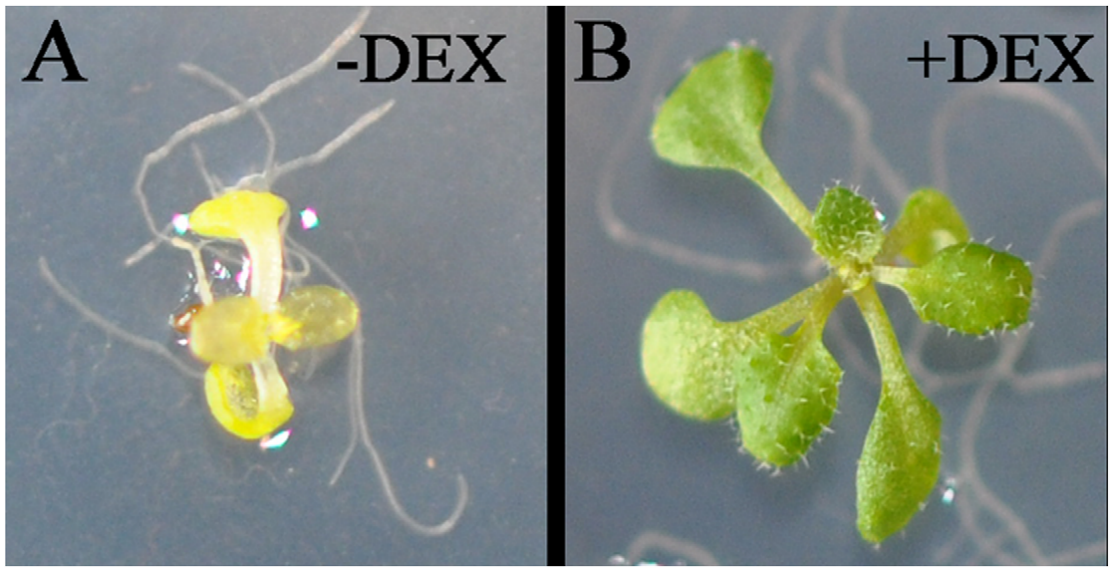
35S::FIT:GR *fit-2* plants express functional FIT protein. Two-week-old plants grown on 1/2 MS in the absence (A) or presence (B) of 30 µM dexamethasone (DEX).

**Figure 2:**
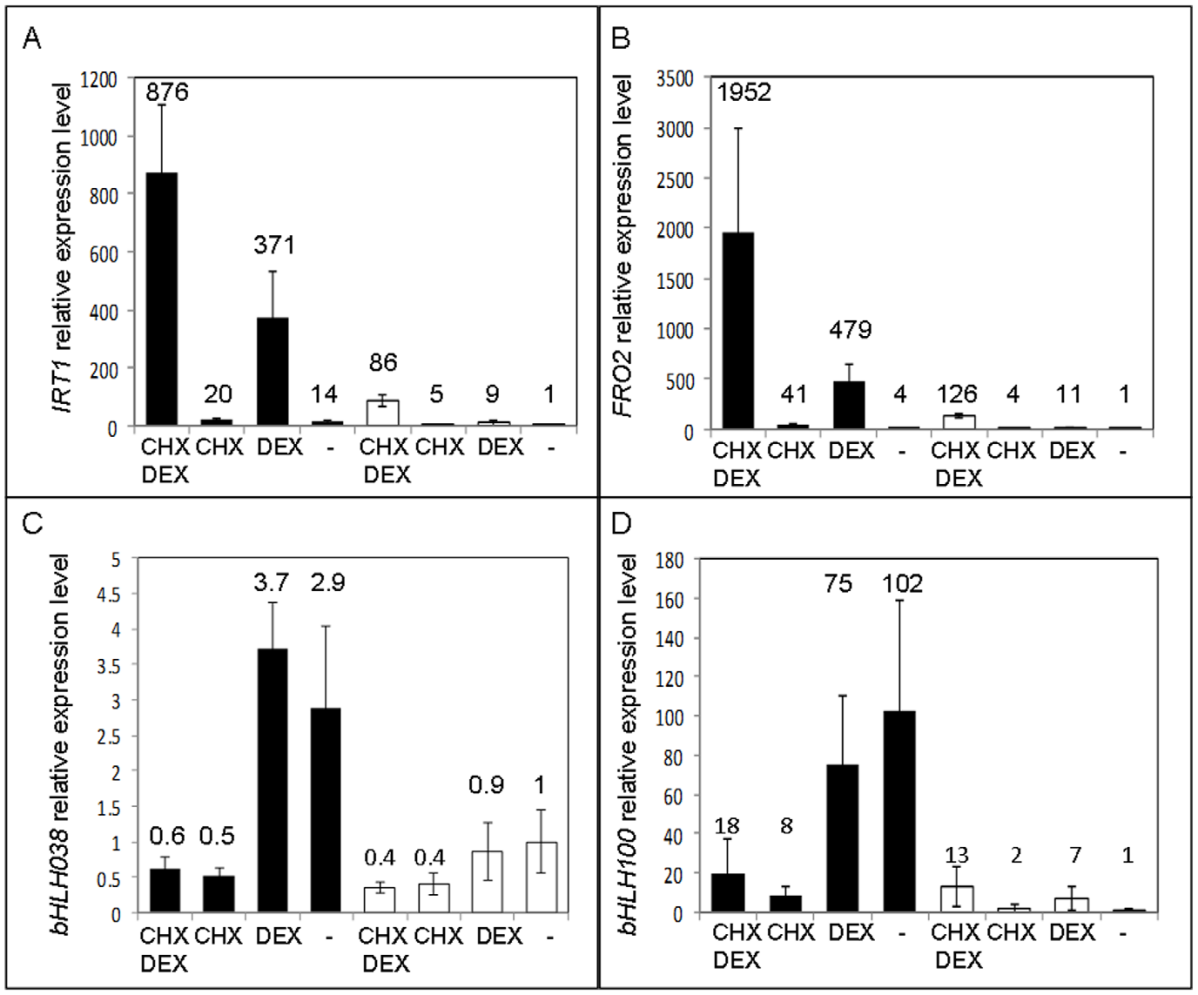
Expression of iron-responsive genes in *fit-2/*35S::FIT:GR plants. 8-day-old plants were grown on 1/2 MS without iron (black) or supplemented with 100 µM Fe (white). Roots were then transferred to liquid media with the same iron content but supplemented as follows: CHX DEX: 100 µM cycloheximide for one hour then 30 µM dexamethasone was added and incubated for 3 hours; CHX: 100 µM cycloheximide for one hour followed by a mock treatment for 3 hours, DEX: mock treated for one hour followed by 30 µM dexamethasone for 3 hours; or mock treatments only. Quantitative RT-PCR results are presented for *IRT1* (A) *FRO2* (B) *bHLH038* (C) and *bHLH100* (D). Error bars indicate standard error (n = 3–4). Relative transcript levels are shown on the graph.

To examine early responses to iron starvation in Arabidopsis that are dependent on the transcription factor FIT, we first determined direct targets of FIT *in vivo*. We generated transgenic *fit-2* knock-out mutant plants expressing a translational fusion between FIT and the rat glucocorticoid receptor (GR), driven by the strong and constitutive 35S promoter (*fit-2*/35S::FIT-GR). In the absence of the GR ligand dexamethasone (DEX), the FIT-GR fusion protein is retained in the cytosol and thus unable to complement the chlorotic phenotype of *fit-2* (Figure 1A). Upon DEX treatment, however, transgenic plants were fully rescued suggesting that the FIT-GR fusion is correctly shuttled to the nucleus and fully functional (Figure 1B). To get a first glimpse of known iron deficiency-regulated genes that are directly controlled by FIT, FIT-GR expressing plants grown in the presence (+Fe) or absence (–Fe) of iron were subjected to short-term mock or DEX treatments. The expression of selected iron-regulated genes was monitored by quantitative RT-PCR. Figure 2A shows that *IRT1* expression is induced by DEX both in the presence and the absence of iron. This is consistent with the hypothesis that FIT is needed to drive *IRT1* expression. To address whether *IRT1* is a direct target of FIT, we preformed the same experiment in the presence of the protein translation inhibitor cycloheximide (CHX). CHX treatment only allows expression of FIT direct target genes, by preventing protein synthesis necessary for transcription of secondary targets. *IRT1* mRNA accumulated in FIT-GR plants supplemented with DEX in the presence of CHX (Figure 2A), indicating that *IRT1* is a direct target of the transcriptional activator FIT.

To date, *IRT1* and *FRO2* have been shown to be co-regulated in response to changes in iron nutrition. We therefore tested whether *FRO2* was also a direct target of FIT. As shown in Figure 2B, *FRO2* gene expression mirrors *IRT1* expression and thus represents a direct target of FIT. Expression of many genes that are misregulated in the *fit-1* knock-down mutant background [4], or simply regulated by iron starvation was then analyzed using the same experimental setup to obtain an overview of the transcriptional network downstream of FIT. In addition to *IRT1* and *FRO2*, *MYB72*, and At3g07720 were found to be directly regulated by FIT (Figure S1). It should be noted that expression of these genes, although dependent on FIT was significantly increased under iron-deficient conditions. Additionally, CHX treatment increased the transcript steady state level of these genes, suggesting that their expression may be under the control of an unstable repressor.

A number of known iron-regulated bHLH transcription factors (bHLH038, bHLH039, bHLH100, bHLH101 and PYE, for example) have been suggested to work directly or indirectly with FIT to drive iron-deficiency responses. However, the molecular mechanisms that control their induction under low iron are still unclear. Here we provide evidence that these genes are not regulated by FIT. Figure 2C and 2D indeed show that *bHLH038* and *bHLH100* are expressed independently of FIT. These two genes are representative of many other known iron-deficiency genes: *NRAMP4*, *ORG1*, *PYE*, *NAS4*, *bHLH039*, and *bHLH101* (Table S1). These genes upregulated by low iron, in a FIT-independent manner, are also likely to be under the control of an unstable factor since their expression is abolished when CHX is present.

Taken together, these data clearly indicate that FIT drives only the expression of a subset of the genes regulated by iron starvation in the root, pointing to the existence of other pathways controlling iron-deficiency responses. A number of transcription factors controlled by iron starvation appear not to be regulated by FIT, such as *PYE*, *bHLH038, bHLH039, bHLH100, bHLH101*, demonstrating the existence of additional regulatory networks driving responses to low iron.

### Identification and Characterization of the *bhlh100/bhlh101* Double Mutant

**Figure 3:**
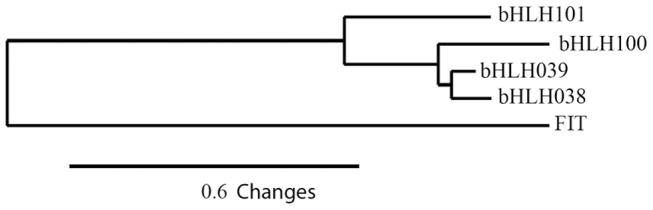
Phylogeny and identification of *bhlh100/bhlh101* double mutant. Phylogenetic tree of closely related bHLH genes involved in the iron-deficiency response. The tree was created using MUSCLE alignment of protein sequences and neighbor joining (bioNJ) on phylogeny.fr [20]–[22].

In order to explore the function of the bHLH transcription factors bHLH100 and bHLH101, we isolated corresponding loss-of-function mutants from publicly available lines for the two genes (SALK_011245 and SALK_074568, respectively). Phylogenetic analysis indicates that *bHLH100* and *bHLH101* cluster together with *bHLH038* and *bHLH039* in a clade of closely related genes within the large bHLH gene family from Arabidopsis (Figure 3). bHLH038 and bHLH039 genes appear in tandem on chromosome 3 and are 79% identical, while sharing 89% similarity. However, bHLH100 and bHLH101 are more distantly related and show only 39% identity and 69% similarity to each other. Previous work has shown that single mutants of the closely related *bHLH038*, *bHLH039*, *bHLH100* and *bHLH101* genes do not have any macroscopic phenotypes [9]. We therefore created the *bhlh100/bhlh101* double mutant. The double mutant for *bHLH100* and *bHLH101* was confirmed not to express wild-type mRNA for either gene (Figure S2). Consistent with previous reports, conditions used in this study demonstrate that both *bHLH100* and *bHLH101* are induced by iron-deficiency in the wild-type background (Figure S2).

### *bhlh100/bhlh101* is Hypersensitive to Iron Deficiency

**Figure 4:**
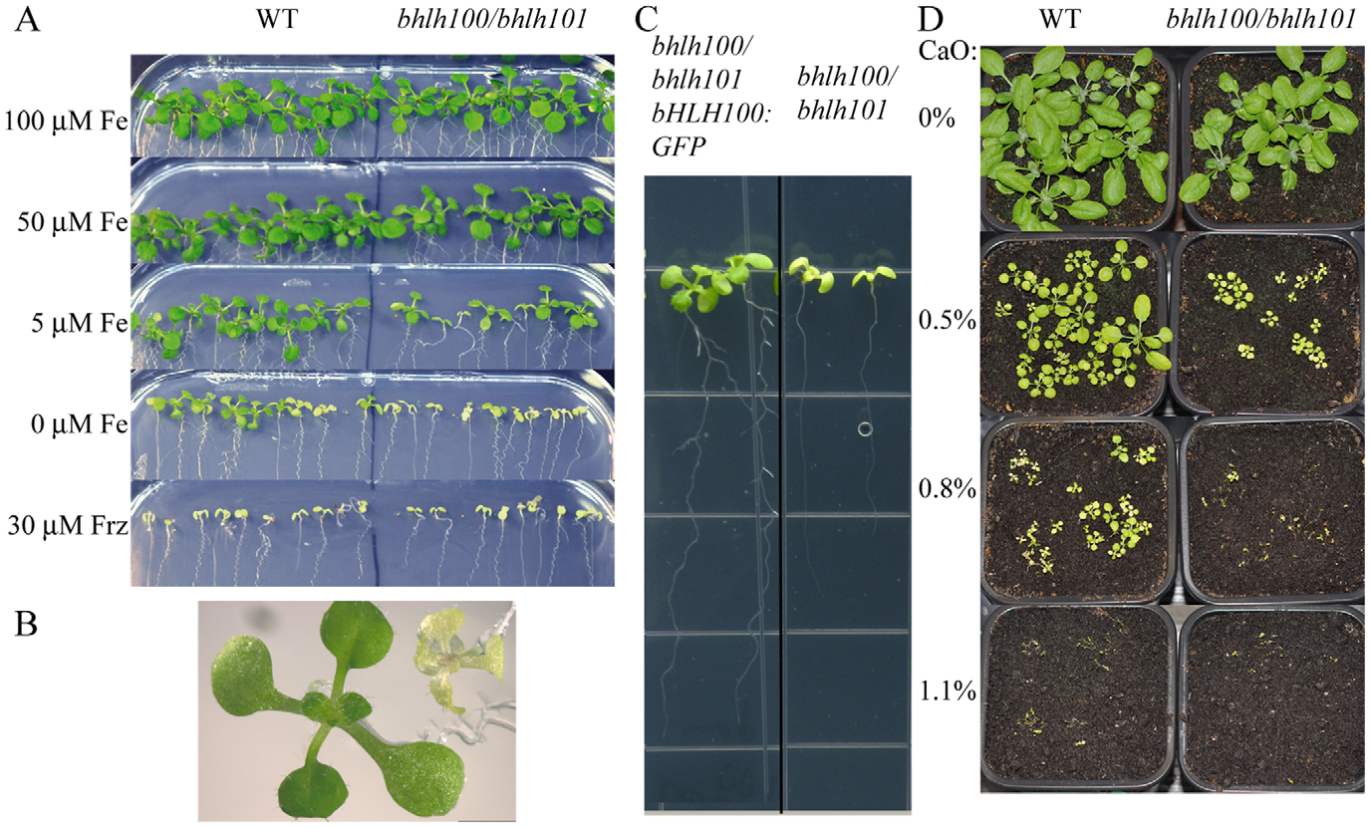
*bhlh100/bhlh101* plants grow poorly on media with low iron as compared to wild-type. (A) 2-week-old wild-type (WT) and *bhlh100/bhlh101* plants were grown on 1/2 MS supplemented with a range of iron concentration or ferrozine (frz) as indicated on the left-hand side. (B) A close-up picture of wild-type (WT, left) and *bhlh100/bhlh101* (right) plants the grown as in (A) on media without added iron. (C) Complementation of low-iron growth defects by expression of a *bHLH100* genomic clone in *bhlh100/bhlh101.* Plants were grown as in (B). (D) 3-week-old wild-type and *bhlh100/bhlh101* plants were grown on soil supplemented with the indicated percent (w/w) of lime (CaO).

To evaluate the potential role of the two iron-starvation induced bHLH transcription factors bHLH100 and bHLH101 in iron homeostasis, we grew wild-type and *bhlh100/bhlh101* double mutant plants on media containing a range of iron concentrations. When iron was present at concentrations of, or above 50 µM, wild-type and the *bhlh100/bhlh101* were indistinguishable (Figure 4A). However at low iron concentrations (no iron added to media), the double mutant was chlorotic and was smaller in size compared to wild-type (Figure 4A–4B). This phenotype was unique to the double mutant and was not observed in either single mutant (Figure S3). When the medium was depleted of iron using the strong iron chelator ferrozine, both wild-type and the *bhlh100/bhlh101* double mutant displayed typical iron-deficiency symptoms including chlorosis and reduced growth (Figure 4A). The chlorotic phenotype of *bhlh100/bhlh101* was rescued by expressing a genomic clone of *bHLH100* in the double mutant background (Figure 4C). The hypersensitivity of the double mutant to low iron in the media was further explored by examining growth on alkaline soil. The high pH of the soil causes a severe reduction in Fe availability for plants. Consistent with what has been observed on iron-deficient medium, wild-type plants displayed chlorotic leaves when grown on soil supplemented with 0.5% (w/w) CaO and near complete inhibition of growth when the soil was supplemented with 1.1% (w/w) CaO (Figure 4D). However, *bhlh100/bhlh101* double mutant was clearly hypersensitive to the CaO and showed growth inhibition at lower concentrations of CaO (0.8% CaO).

**Figure 5:**
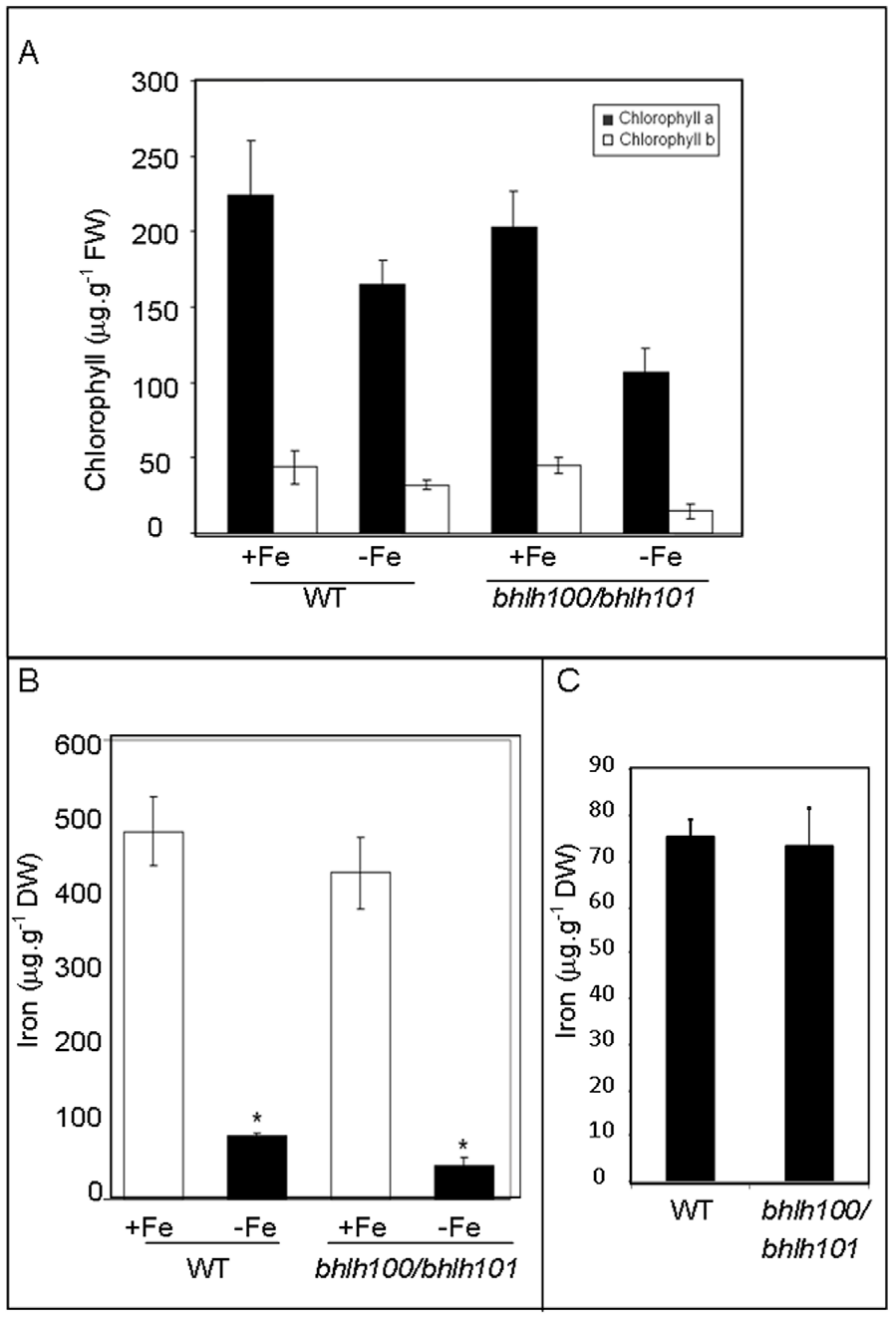
*bhlh100/bhlh101* plants accumulate less chlorophyll and less iron than wild-type. (A) Chlorophyll content expressed per gram of fresh weight (FW) was measured in extracts of plants grown on 1/2 MS without added iron (–Fe) or supplemented with 100 µM Fe (+Fe). (B) Iron was measured in plants grown on 1/2 MS without added iron (–Fe) or in the presence of 100 µM Fe (+Fe). * indicates a statistically significant difference between wild-type (WT) and *bhlh100/bhlh101*. (C) Seed iron content of wild-type (WT) and *bhlh100/bhlh101*.

A hallmark of iron deficiency in plants is reduced chlorophyll content. Chlorophyll content for wild-type and double mutant plants grown both under iron-sufficient and iron-deficient conditions was examined. *bhlh100/bhlh101* double mutant and wild-type plants harbored comparable chlorophyll levels under iron-sufficient conditions, while the double mutant had significantly less chlorophyll under iron-deficient conditions (Figure 5A; p-value = 0.033 for chlorophyll b, and p-value = 0.055 for chlorophyll a). Together this suggests that under conditions of low iron, the double mutant does not accumulate enough iron to assemble the normal photosynthetic apparatus.

### Iron Content of *bhlh100/bhlh101*

To decipher if the chlorotic phenotype displayed by the *bhlh100/101* double mutant under low-iron conditions may be explained by a lack of iron in the plant, we determined the iron content of 10-day-old wild-type and double mutant plants. Wild-type and double mutant plants grown under iron-sufficient conditions showed no difference in total iron content when grown in the presence of iron (Figure 5B), consistent with the lack of phenotypes under such growth conditions. However, when iron was omitted from the medium, the double mutant accumulated approximately half as much iron in its tissues than wild-type plants (Figure 5B). The impaired growth of the *bhlh100/bhlh101* double mutant is not explained by lower iron content in the seed. Wild-type and *bhlh100/bhlh101* double mutant seeds indeed contained 75.2±2.3 µg.g^−1^ and 73.2±4.9 µg.g^−1^ of iron, respectively (n = 3; Figure 5C). Consistent with the absence of phenotype under low iron conditions, the *bhlh100* and *bhlh101* single mutants showed also no difference in biomass production (Figure S3A). Altogether, these results suggest that bHLH100 and bHLH101 are important regulators acting redundantly to control iron homeostasis.

### *bhlh100/bhlh101* Double Mutant Plants are Late Flowering

**Figure 6:**
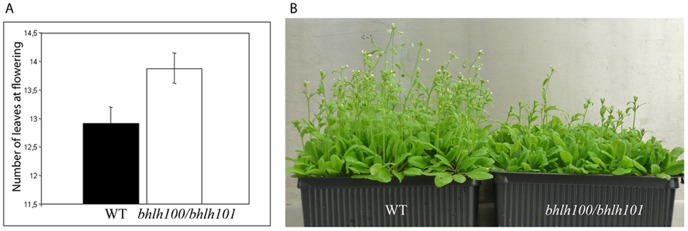
*bhlh100/bhlh101* plants flower later than wild-type. (A) Number of leaves of greenhouse-grown wild-type (WT) and *bhlh100/bhlh101* double mutant plants. Leaves were counted at the time of bolting (n = 25 for each genotype; p-value = 0.0170). (B) Greenhouse-grown plants displaying late-flowering phenotype.

Besides the dramatic hypersensitivity to iron deficiency, *bhlh100/bhlh101* also displayed a late flowering phenotype (Figure 6). On average, the *bhlh100/bhlh101* double mutant began bolting with one more leaf than wild-type plants. This phenotype appears to be independent of iron in the soil since plants watered with excess iron exogenously still flowered late (Figure S4). Interestingly, while iron treatment did result in an earlier flowering time for both the wild-type and the double mutant, the difference in flowering time between the two genotypes remained.

### *bHLH100* and *bHLH101* do not Regulate FIT Direct Targets

To gain some insights into the biological role of bHLH100 and bHLH101, we first examined if the direct targets of FIT were misregulated in the *bhlh100/bhlh101* double mutant background. bHLH100 and bHLH101 were indeed proposed to act similarly to their bHLH038 and bHLH039 counterparts that directly interact with FIT to drive expression of *IRT1* and presumably other FIT target genes [11].

**Figure 7:**
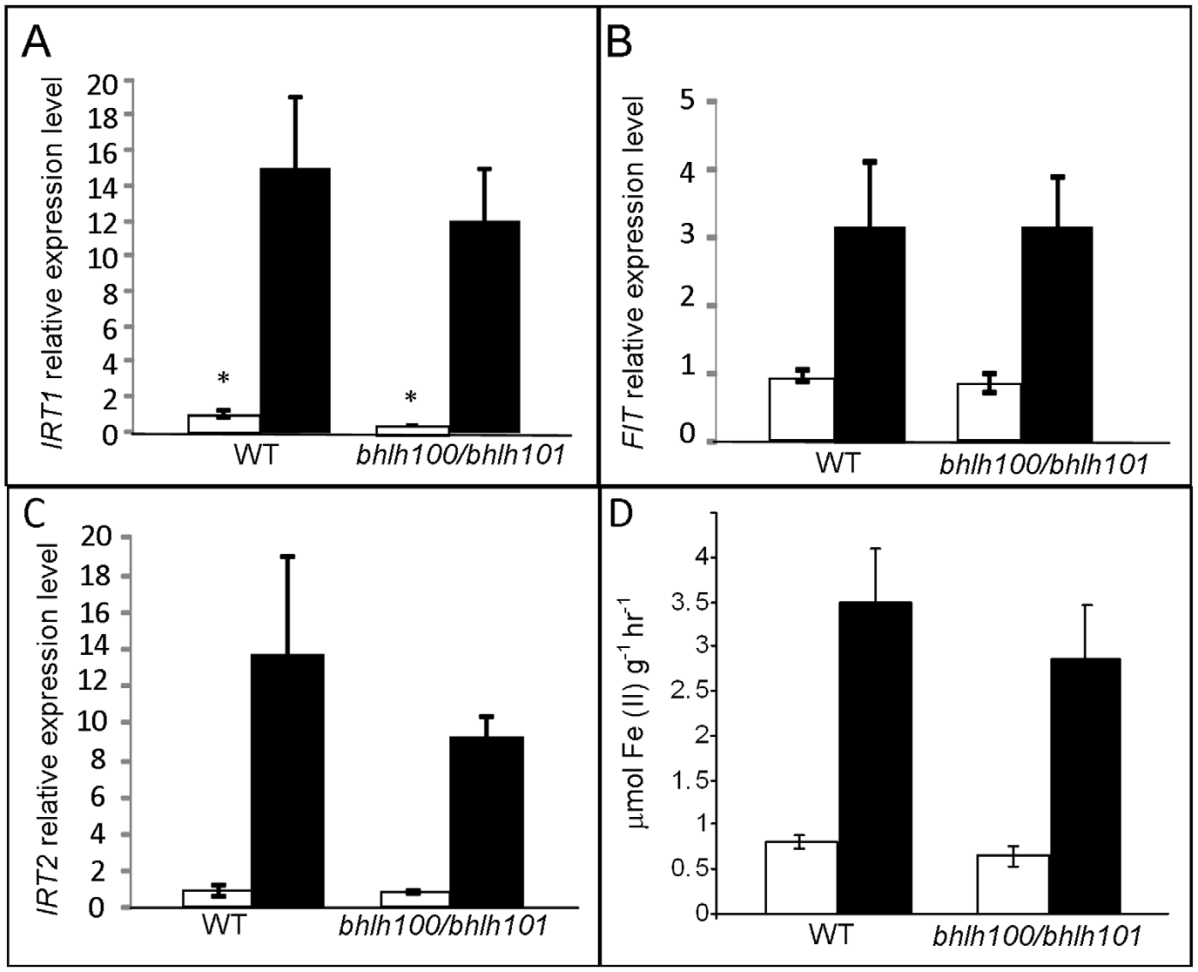
Expression and activity of selected FIT direct targets in *bhlh100/bhlh101* and wild-type backgrounds. Relative gene expression level measured by quantitative RT-PCR of *IRT1* (A), *FIT* (B), and *IRT2* (C) in wild-type (WT) and *bhlh100/bhlh101* plants grown on 1/2 MS without iron (black) or in the presence of 100 µM Fe (white). (D) Root ferric reductase activity from WT and *bhlh100/bhlh101* plants grown as in A–C. Error bars show standard error (n = 3–4). * indicates a statistically significant difference (p-value <0.05) between wild-type and *bhlh100/101* for the indicated condition.

Using quantitative RT-PCR, we examined the expression of several FIT direct targets and *FIT* itself in the *bhlh100/bhlh101* double mutant background. As seen in Figure 7, *IRT1, IRT2* and *FIT* expression was induced by low iron similarly in both wild-type and the double mutant. We did notice a slight, but significant increase in *IRT1* expression under iron-sufficient conditions. This increased *IRT1* expression level is still dramatically lower than levels attained under conditions of iron-deficiency, and is not reflected at the IRT1 protein level as observed by western blot (Figure S5). We also investigated root ferric reductase activity in wild-type and *bhlh100/bhlh101* plants, but observed no change in activity in the double mutant (Figure 7D). These observations argue for a FIT-independent role of bHLH100 and bHLH101 in directing iron homeostasis.

**Figure 8:**
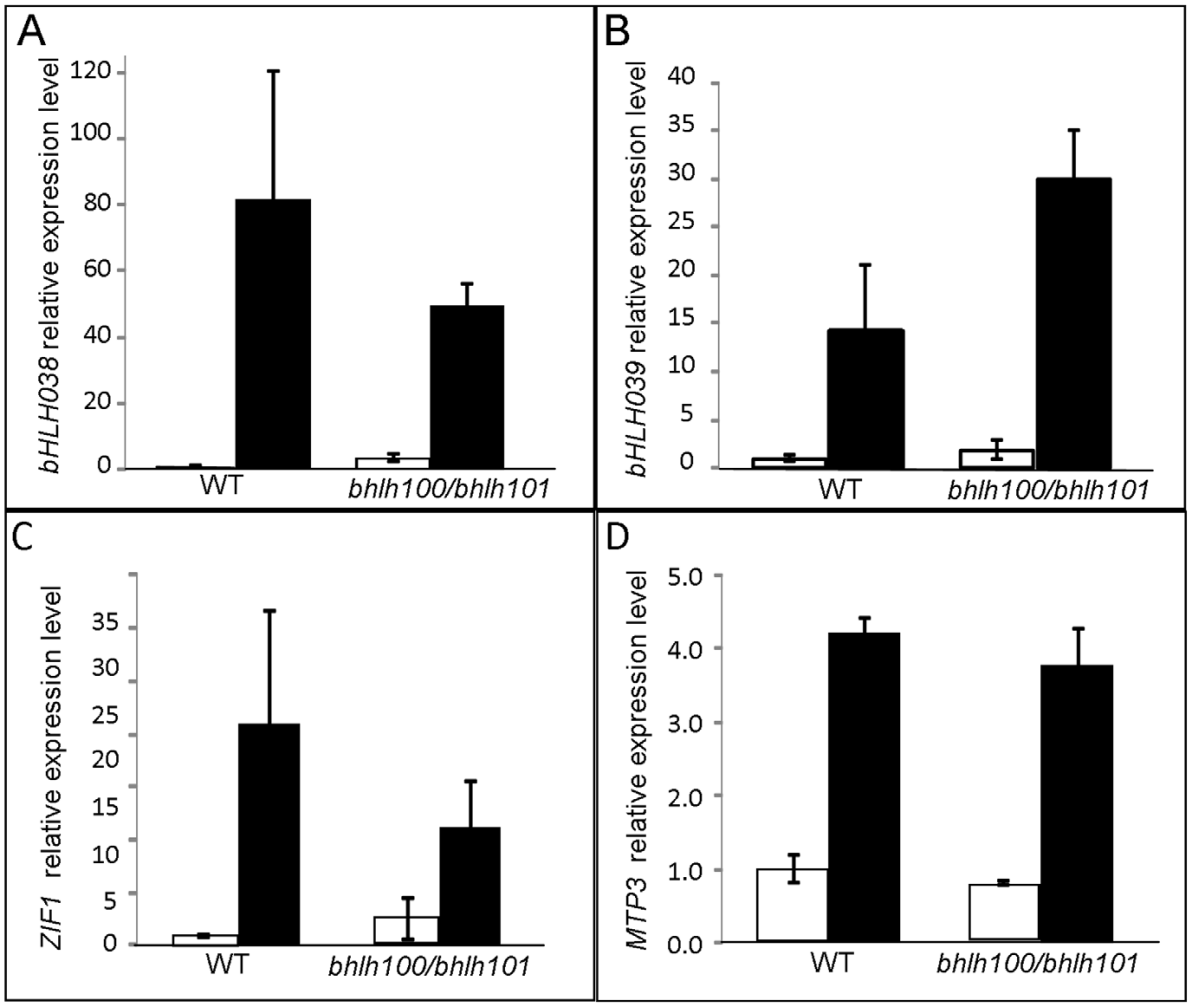
Expression of selected FIT-independent genes in *bhlh100/bhlh101* and wild-type backgrounds. Relative gene expression level of *bHLH038* (A), *bHLH039* (B), *ZIF* (C), and *MTP3* (D) in wildtype (WT) and *bhlh100/bhlh101* plants grown on 1/2 MS without iron (black) or in the presence of 100 µM Fe (white). Error bars indicate standard error (n = 3–4).

To shed light on the gene networks under the control of bHLH100 and bHLH101, we extended our analysis to iron-regulated genes that are not under the control of FIT. Figure 8 shows the expression *of bHLH038, bHLH039, ZIF1* and *MTP3* in wild-type and *bhlh100/bhlh101*. Again, none of the candidate genes investigated were shown to be significantly deregulated in the double mutant background. These four genes are known to play a role in iron-deficiency responses but are not direct targets of FIT. These negative results suggest that bHLH100 and bHLH101 regulate responses to iron-deficiency in a yet undiscovered pathway.

### Genome-wide Gene Expression Analysis

To obtain a comprehensive view of the genes misregulated in the *bhlh100/bhlh101* double mutant background, we performed whole-genome gene expression analysis using ATH1 affymetrix microarrays. Roots and shoots of 10-day-old plants, as in Figure 5, grown either without iron or supplemented with 100 µM iron were harvested separately for microarray analysis. Consistent with previous reports of gene expression changes under iron-deficiency, wild-type plants showed a typical induction of iron-deficiency-responsive genes like *IRT1*, *IRT2*, *MYB10*, *MYB72*, *bHLH39*, *PYE*, *OPT3* in roots (Table S1). Specifically, *bHLH101* showed the greatest fold change in response to iron deprivation in roots with a 30-fold increase in mRNA accumulation. In shoots *bHLH101* was induced approximately 5-fold by iron deficiency in wild-type plants. The expression of *bHLH100* cannot be monitored since no corresponding probes are found on the ATH1 Genome Array. The expression of *bHLH101* was completely abolished in the *bhlh100/bhlh101* double mutant, consistent with the lack of mRNA observed by quantitative RT-PCR (Table S1 and Figure S2).

**Figure 9:**
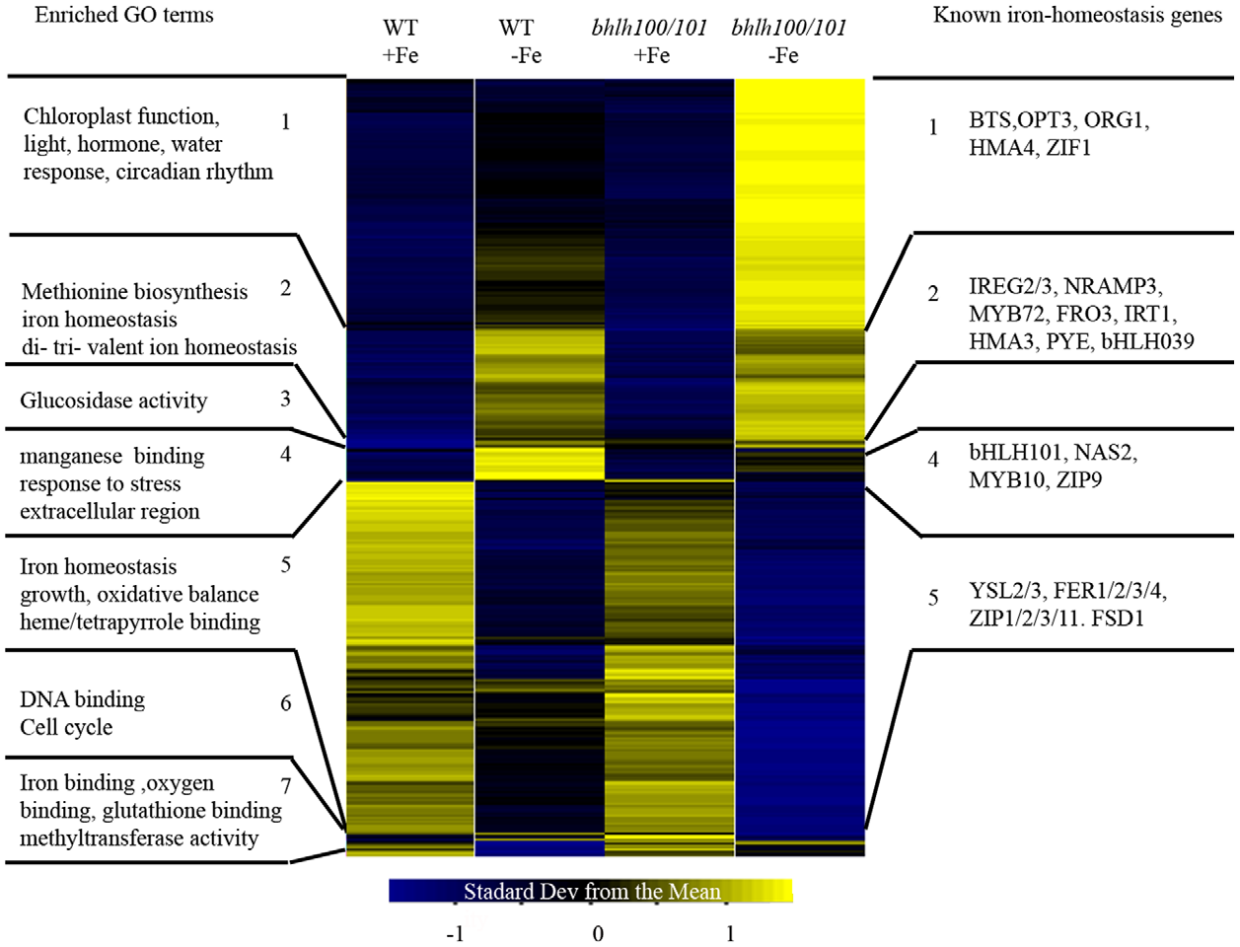
Heatmap of differentially expressed genes in roots of wild-type and *bhlh100/bhlh101* plants. Genes found to be differentially expressed between the +Fe conditions (100 µM Fe) and the –Fe conditions for both wild-type (WT) and *bhlh100/bhlh101* plants were clustered using a Pearson correlation into 12 clusters. Clusters with significantly enriched GO terms (left) or known iron-regulated genes (right) were numbered.

**Figure 10:**
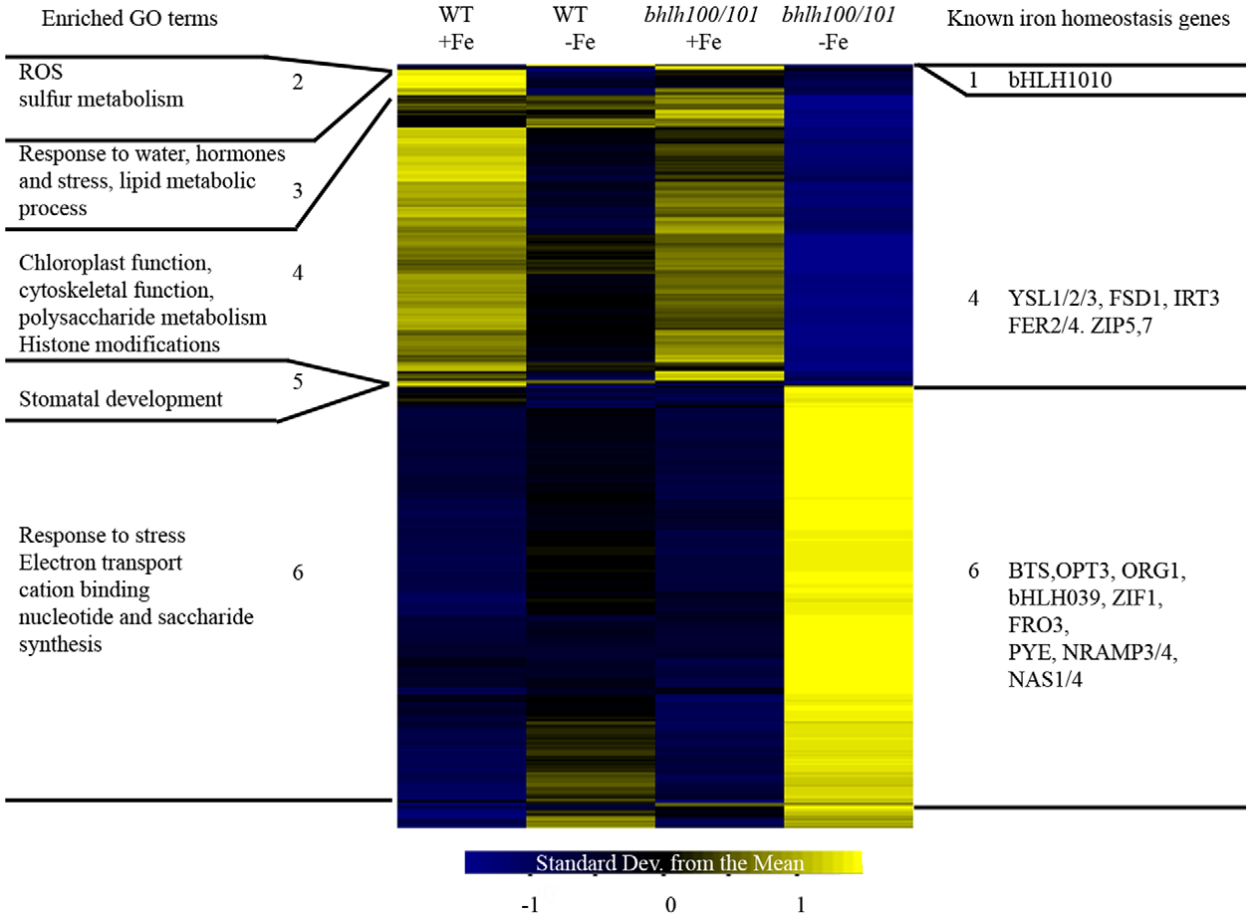
Heatmap of differentially expressed genes in shoots of wild-type and *bhlh100/bhlh101* plants. Genes found to be differentially expressed between the +Fe conditions (100 µM Fe) and the –Fe conditions for both wildt-ype (WT) and *bhlh100/bhlh101* plants were clustered using a Pearson correlation into 12 clusters. Clusters with significantly enriched GO terms (left) or known iron-regulated genes (right) were numbered.

To better highlight the network of iron-responsive genes regulated by both bHLH100 and bHLH101, we performed hierarchical clustering analysis. Genes with expression changes that were both statistically significant (p-value<0.05) and that were at least 1.5 fold induced or repressed by low iron (Table S1) were clustered using a Pearson correlation. Statistical analyses revealed the existence of 2502 and 2289 genes with altered expression in roots and shoots, respectively (Figure 9, 10). The first observation is that the genomic signature of *bhlh100/bhlh101* clearly argues for a strong defect in iron homeostasis with many iron deficiency-induced genes being upregulated to a stronger extent in *bhlh100/bhlh101.* These include *OPT3*, *BTS* and *ORG1* in both roots and shoots, and *PYE*, *NRAMP3/4*, *FRO3*, *bHLH39* in shoots specifically (Figures 9, 10 and Table S2). Conversely, genes that are induced by iron toxicity or down-regulated by iron starvation (*AtFer1-4*, *YSL1-3*) in wild-type plants clearly show exacerbated repression in *bhlh100/bhlh101.*

Analysis of the clusters in roots indicates that FIT direct targets (*IRT1* and *MYB72*) and genes regulated independently of FIT (*PYE* and *bHLH039*) are induced by low iron equally in both the wild-type and the double mutant (Figure 9, Cluster 2). This further supports the idea that bHLH100 and bHLH101 act independently of FIT. Other FIT-independent genes such as *BTS*, *ORG1* and *OPT3* form a separate cluster (cluster 1) that is induced by low iron, but this induction is stronger in the double mutant than in wild-type plants. We also found a large group of genes (cluster 5) repressed by low iron both in the wild-type and the double mutant. More interesting is cluster 4, which contains 97 genes induced by low iron in wild-type but not induced in the *bhlh100/bhlh101* double mutant (Table S3). Cluster 4 notably comprises known iron-responsive genes such as *NAS2*, *ZIP9*, *MYB10*, *CYP71B5*, and *CYP82C4*.

In the shoots, cluster 6 shows genes more strongly up-regulated by low iron in the double mutant and contains known iron-regulated genes like *BTS*, *OPT3*, *ORG1*, *bHLH039*, *ZIF1*, *FRO3*, *PYE*, *NRAMP3/4*, *NAS1/4* (Figure 10). These genes are all FIT-independent, which is expected since FIT is not active in the shoots. Cluster 1 contains the genes that are induced by low iron in the wild-type but not in the double mutant, like *bHLH101*. This cluster only contains 11 genes with no enriched GO terms and no other known iron-responsive genes (Table S3). Altogether, these observations point to the strict requirement for bHLH100 and bHLH101 for genome-wide reprogramming in response to iron starvation in both roots and shoots, and highlight the gene networks controlled by bHLH100 and bHLH101 in both roots and shoots.

## Discussion

It is becoming increasingly clear that iron-homeostasis in plants is regulated by a complex set of pathways, with different tissues harboring different responses [12]. FIT remains the master regulator of root iron-uptake responses, but other pathways are emerging that likely control other responses to iron deficiency such as developmental changes, internal redistribution of iron within the plant or organelles, control of ionic balances between the various divalently charged transition metals (i.e. Fe, Mn, Zn, Co, Mo), etc. A number of bHLH transcription factors have been involved in (FIT, PYE), or loosely associated with (bHLH038/039/100/101) iron-deficiency responses. Here we provide a better picture of the gene networks controlled by the master regulator FIT and show that bHLH100 and bHLH101, although critical for proper responses to iron starvation, act independently of FIT. This is in contrast to their closely related homologs, bHLH038 and bHLH039, which have been suggested to work in concert with FIT in the control of *IRT1* and *FRO2* gene expression [11].

Using transgenic plants expressing an inducible version of FIT (FIT:GR), we determined direct targets of FIT among a long list of known iron-regulated genes. These include IRT1, FRO2, and MYB72. Although IRT1 is clearly a direct target of FIT (Figure 2A), IRT1 also appears to be under the control of a rapidly-turned over repressor since IRT1 is upregulated when protein translation is inhibited by CHX, likely to keep IRT1 in check and avoid spurious expression. Additionally, we see that low iron results in increased expression of IRT1, again indicating a role for some protein other than FIT present only under iron-deficient conditions. This factor that moderates IRT1 transcription under low iron has not yet been identified. This factor is unlikely to be bHLH038 or bHLH39 since they are not expressed during the conditions of CHX treatment (Figure 2C). Clearly identification and characterization of the CHX sensitive and low-iron-regulated elements directing iron-uptake would be of great interest.

A set of root and shoot expressed genes that are regulated independently of FIT (Figure 2 and Figure S1) was also identified. The iron-regulated, FIT-independent genes, with the exception of BTS, PYE, IRT3, and FRD3, showed an expression pattern that was induced only in the absence of CHX (Figure S1). This suggests that a rapidly turned over protein is important for controlling iron-deficiency responses. However, in this particular case the rapidly turned over protein seems to be positively regulating FIT-independent processes. BTS and PYE were not affected by CHX presence and therefore are independent of the one or several rapidly turned over elements. Intriguingly, we did not find genes regulated indirectly by FIT, meaning induced by dexamethasone but not in the presence of both cycloheximide and dexamethasone. This may suggest that FIT is the very downstream factor in the pathway leading to the activation of iron uptake from soil. However, since MYB72 was identified as a direct target and is a putative transcription factor, we would expect that FIT has indirect targets that must pass through MYB72. In sum, this study shows that many signals are integrated to give rise to iron-deficiency responses: FIT-dependent, FIT-independent, CHX-dependent, CHX-independent, and iron-dependent.

In order to explore more thoroughly the FIT-independent pathways regulating iron homeostasis, a double mutant of two FIT-independent genes *bHLH100* and *bHLH101* was created. These double mutant plants appear to be defective in growth on low iron (Figure 4), and under these conditions accumulate less chlorophyll and less iron (Figure 5),yet the majority of known iron-deficiency genes were expressed in a similar fashion as in the wild-type plants. The fact that *IRT1* and *FRO2* are not upregulated in the *bhlh100/bhlh101* mutant background is striking considering the dramatic chlorosis and decreased iron content of *bhlh100/bhlh101* plants. A few hypotheses could explain the defect in iron accumulation of *bhlh100/bhlh101* considering that the expression of the root iron machinery gene is intact. A first possibility is that bHLH100 and bHLH101 control root acidification, a prerequisite to root iron uptake, but no *AHA* genes were found misregulated in our genome-wide gene expression survey of *bhlh100/bhlh101*. The second scenario relies on bHLH100 and bHLH101 acting post-translationally to regulate the root iron uptake machinery. However, we observed wild-type accumulation of IRT1 protein and root ferric reductase activity in *bhlh100/bhlh101* (Figures 7D and S5), strongly suggesting that the defects of the double mutant are not caused by impaired iron uptake. Most likely, the phenotype of *bhlh100/bhlh101* double mutant may be explained by defects at other levels of control of iron homeostasis such as the chelation, the distribution between tissues and organelles, the utilization and the storage of iron. Alternatively, bHLH100 and bHLH101 may be responsible for the plant’s adapted growth to low-iron conditions. This hypothesis is supported by the fact that wild-type but not the double mutant were able to become green and grow on severely iron-deficient media.

Microarray data suggests that bHLH100 and bHLH101 are important for regulating growth on low iron media by directing remobilization and adapted growth. Figure 9 and Table S3 show that *NAS2*, *CYP71B5*, *CYP82C4*, *MYB10*, and *ZIP9* were all significantly induced by low iron in wild-type roots but this induction was almost abolished in the *bhlh100/bhlh101* double mutant. NAS2 is involved in nicotianamine synthesis. Nicotianamine is a metal ligand thought to help shuttle iron within the plant, as evidenced by the severe chlorosis observed in a quadruple mutant of Arabidopsis lacking NAS1-4 function [13]. It is possible that the defects observed in *bhlh100/bhlh101* plants are due to the iron not reaching the location (cell or organelle) where it is needed because there is a lack of ligand availability. CYP71B5 and CYP82C4 are cytochrome P450 enzymes involved in the iron-deficiency responses [14]. The loss of theses enzymes could be a symptom of iron not reaching its target as they require a heme moiety to function or their loss could result in a plant that is unable to cope with low iron conditions. ZIP9 is a transporter likely to function in moving metals (zinc and possibly iron) around in the plant, and MYB10 is a putative transcription factor that is thought to direct iron homeostasis [4]. However, no functional data is available to date on the role of ZIP9 or MYB10 in plants. These genes together likely mediate the re-distribution of iron and other divalent ions within the plant, as part of the plant response or adaptation to iron deficiency. Thus bHLH100 and bHLH101 regulate the low-iron induction of these genes to maintain growth under conditions of low iron. In the shoots, the function of bHLH100 and bHLH101 is less clear. Only a few genes, so far unrelated to iron homeostasis, were not induced by low iron in the *bhlh100/bhlh101* double mutant (Table S3). Most of these genes have only predicted or unknown functions. Among them were found genes encoding a beta-amylase, a a putative sucrose phosphate synthase, and a subunit of photosystem I. Such genes are likely required for optimal metabolism and growth under low iron conditions, and lack of induction may explain in part the small chlorotic phenotype of *bhlh100/bhlh101* plants. It should be noted that many genes were induced to a greater extent by low iron in the double mutant. This hyper-induction of genes might be a secondary effect to the double mutant plant’s inability to redistribute metals internally and harboring low shoot iron content. Conversely, we cannot rule out the possibility that this hyper-induction is actually a direct consequence of the loss of bHLH100 and bHLH101 and thus serves to curb overexpression of key genes.

Altogether, we provide evidence for the existence of additional transcriptional networks directing iron-deficiency responses in plants. bHLH100 and bHLH101 appear to function redundantly, although independently of FIT, to direct metabolic changes within the plant in response to low iron. We favor the hypothesis that these genes control redistribution of iron within the plant as evidenced by their control over several genes involved in the re-distribution of iron. However, we should note that although iron-uptake responses such as the expression of *IRT1* and *FRO2* are unchanged in the double mutant, there is a reduction of iron in the plant. This could reflect a partial uncoupling of sensing of the iron deficiency status of the plant and the root response suggesting that bHLH100 and bHLH101 function to fine-tune the iron-deficiency response signaling. Of particular interest is the identification of factors controlling the iron-regulated expression of *bHLH100* and *bHLH101*, and to better understand how iron is sensed and how this status is communicated to all plant tissues.

## Materials and Methods

### Constructs

*FIT* cDNA was amplified by RT-PCR on RNA extracted from young seedlings using FIT F and FIT R primers (Table S4), and cloned in pCHF3 at the BamHI-SalI sites, downstream of the CaMV35S promoter. The regulatory domain from the rat glucocorticoid receptor was amplified from pRS20 using the GR F and GR R primers, and cloned in pCHF3-FIT at the SalI site in frame with *FIT*. The genomic region of *bHLH100* was amplified by PCR using the following primers bHLH100 F and bHLH100 R, subcloned in pCR8/GW/TOPO (Invitrogen), before recombination in pGWB4 [15].

The different constructs were transferred to *Agrobacterium tumefaciens* strain GV3101, and transformed into *fit-2* or *bhlh100/bhlh101* plants by floral dipping [16].

### Growth Conditions

Plants were grown in sterile conditions on vertical plates at 21°C with 16 hours of light unless otherwise noted. Seeds were surface-sterilized and sown on half-strength Murashige and Skoog medium (1/2 MS) containing 1% sucrose and 1% agar. The medium was buffered with 0.5 g.L^−1^ 2-N-morpholino-ethanesulfonic acid (MES), and the pH was adjusted to 5.7 by KOH, and supplemented with FeEDTA. Low iron conditions corresponded to omission of FeEDTA in the medium whereas iron-deficient conditions were achieved by addition of 30 µM Ferrozine [3-(2-pyridyl)-5,6-diphenyl-1,2,4-triazine sulfonate], a strong iron chelator.

For chemical treatments, roots were excised and transferred to liquid media with the same iron content. Roots were subjected to a one-hour incubation in the presence of mock or 100 µM CHX, followed by a 3 hour treatment with either mock or 30 µM DEX.

### Isolation of *bhlh100/bhlh101* Double Mutant

T-DNA mutant lines SALK_074568 and SALK_011245, corresponding to *bHLH100* (At2g41240) and *bHLH101* (At5g04150) respectively, were genotyped using the primers LB2, SALK_074568 F, bHLH100 R, SALK_011245 F and SALK_011245 R (Table S4). Homozygous single mutants were crossed and the F2 progeny genotyped for *bhlh100/bhlh101* double homozygous plants.

### Chlorophyll Content

Chlorophyll was extracted from plants by adding 1% HCl in ethanol (v/v) (100 µl.mg^−1^ tissue). The absorbance of samples was measured at 645 and 663 nm, respectively. The following equations were used to calculate chlorophyll content: Chlorophyll a = −5.200*A645+13.528*A663; Chlorophyll b = 22.433*A645–7.074*A663 [17].

### Iron Content

The iron concentration in leaves was measured as described previously [18]. Iron content was determined using a standard curve made from a dilution series of FeSO_4_.

### RNA Preparation and Quantitative RT-PCR

Total RNA was extracted using the RNeasy kit (Qiagen) using on column digestion of genomic DNA with DNAse (Qiagen). cDNAs were prepared following manufacturer’s instructions using M-MLV Reverse Transcriptase Rnase H minus (Promega) and oligo(dT)15 primer (Promega).

Semi-quantitative RT-PCR was performed to analyze transcript accumulation in wild-type and *bhlh100/bhlh101* double mutant plants (26 cycles). Real-time quantitative RT-PCR was performed using a LightCycler480 with Fast-Start DNA MasterPLUS SYBER GREEN I (Roche Applied Science, www.roche-applied-science.com) and gene-specific primers (Table S4). Analysis of relative transcript accumulation was done using LightCycler480 SW1.5. Relative expression level was determined using the housekeeping gene AP-4 complex subunit mu-1 (At4g24550), a protein member of the clathrin complex. Quantitative RT-PCR was performed using three independent biological replicates and three technical replicates of each biological sample.

### Microarray Analysis

RNA was extracted as described above and amplified using 3'IVT Express kit (Affymetrix). 15 µg of amplified, fragmented, and biotin labeled antisense RNA were hybridized to ATH1 arrays. Arrays were scanned using the GeneChip® Scanner 3000 7G (Affymetrix). All experiments were done in triplicates. Microarray data were deposited at Gene Expression Omnibus (http://www.ncbi.nlm.nih.gov/geo; number GSE40076). Data manipulations were performed using R (http://www.r.project.org) and was normalized using RMA. Significant genes were found by taking a p-value of <0.05 and a fold change value of either 1.5. Clustering was done using a Pearson correlation and a complete method for aggregation. Enriched GO terms were found using VirtualPlant [19].

## Supporting Information

Figure S1 **Expression of selected iron-responsive genes in** ***fit-2/*** **35S::FIT:GR plants.** 8-day-old plants were grown on 1/2 MS without iron (black) or supplemented with 100 µM Fe (white). Roots were then transferred to liquid media with the same iron content but supplemented as follows: CHX DEX: 100 µM cycloheximide for one hour then 30 µM dexamethasone was added and incubated for 3 hours; CHX: 100 µM cycloheximide for one hour followed by a mock treatement for 3 hours, DEX: mock treated for one hour followed by 30 µM dexamethasone for 3 hours; or mock treatments only. Quantitative RT-PCR results are presented for with each gene’s relative expression level (REL) as indicated. When standard error bars are present n = 3–4.
(TIF)

Figure S2 ***bhlh100/bhlh101*** **plants do not express** ***bHLH100*** **or** ***bHLH101.*** Quantitative RT-PCR using primers specific for *bHLH101* (A) and *bHLH100* (B) performed on total RNA extracted from wild-type (WT) and double mutant plants (n = 3). Plants were grown either in the absence of iron (black bars) or presence of iron (white bars; n = 3).
(TIF)

Figure S3 ***bhlh100*** **and** ***bhlh101*** **single mutants do not display growth defects on low iron.** 3-week-old wild-type (WT), *bhlh100*, *bhlh101*, and *bhlh100/bhlh101* were grown on 1/2 MS plates without added iron (A) or with 100 µM Fe (B). The biomass of wild-type, *bhlh100*, *bhlh101* and *bhlh100/bhlh101* plants grow under iron-limited conditions (A) is shown.
(TIF)

Figure S4 **Flowering time of wild-type and** ***bhlh100/bhlh101*** **plants supplemented with iron.** *bhlh100/bhlh101* plants flower late irrespective of iron nutrition. Plants were grown in the greenhouse and irrigated with either sequestrene (+Fe) or water from the start of cultivation. Leaves were counted at the time of flowering. For both watering treatments, wild-type plants (WT, black) had significantly less leaves at time of flowering than the double mutant (white) (p-values of 0.0098 and 0.0272 respectively).
(TIF)

Figure S5 **IRT1 protein accumulates to the same level in both wild-type and** ***bhlh100/bhlh101*** **plants.** Western blot of total protein extracts from 10-day-old wild-type (WT) and *bhlh100/bhlh101* roots grown on 1/2 MS without added iron (–Fe) or with 100 µM Fe (+Fe). * indicates non-specific band is used as loading control.
(TIF)

Table S1 **Expression values for plants grown on +Fe or –Fe. The first worksheet contains values for wild-type plants, while the second contains values for** ***bhlh100/bhlh101.*** The AffyID, unique identifier (ACCNUM), gene symbol, and description of each gene are given. Average logged (base 2) values of hybridization to each ATH1 probe are given. Three biological replicates were extracted from root and shoot tissues of 10-day-old plants grown on 1/2 MS with or without 100 µM Fe.
(XLSX)

Table S2 **Array data from wild-type and** ***bhlh100/bhlh101*** **roots and shoots grown with or without iron. The first worksheet contains data from plants grown in the presence of 100 µM Fe, and the second is data from plants grown in the absence of iron.** The AffyID, unique identifier (ACCNUM), gene symbol, and description of gene are given. Average logged (base 2) values of each probe are given for root tissues from the double *bhlh100/bhlh101* mutant (dm) and wild-type (WT) are listed in columns “Roots dm” and “Roots WT” respectively. The fold change between these two is given in column “fc.dm/wt.” “tt.dmwt0.”gives the p-value for this fold change. Average logged (base 2) values of each probe are given for shoot tissues from the double *bhlh100/bhlh101* mutant (dm) and wild-type (WT) are listed in columns “Shoots dm” and “Shoots WT” respectively. The fold change between these two is given in column “fc.dm/wts.” “tt.dmwt0s.” gives the p-value for this fold change.
(XLSX)

Table S3 **Root- and shoot-expressed genes that cluster with** ***bHLH101*** **.** The AffyID, unique identifier (ACCNUM), gene symbol, and description of each gene are given. Average logged (base 2) values of hybridization to each ATH1 probe are given.
(XLSX)

Table S4 **Primers used in this study.** A list of primers used for cloning, genotyping and quantitative RT-PCR in this study.
(XLSX)
