# Supplementary figures and images for: Arabidopsis bHLH100 and bHLH101 Control Iron Homeostasis via a FIT-Independent Pathway
Source: PLoS One. 2012 Sep 11;7(9):e44843. doi: 10.1371/journal.pone.0044843 (PMC3439455; doi:10.1371/journal.pone.0044843)

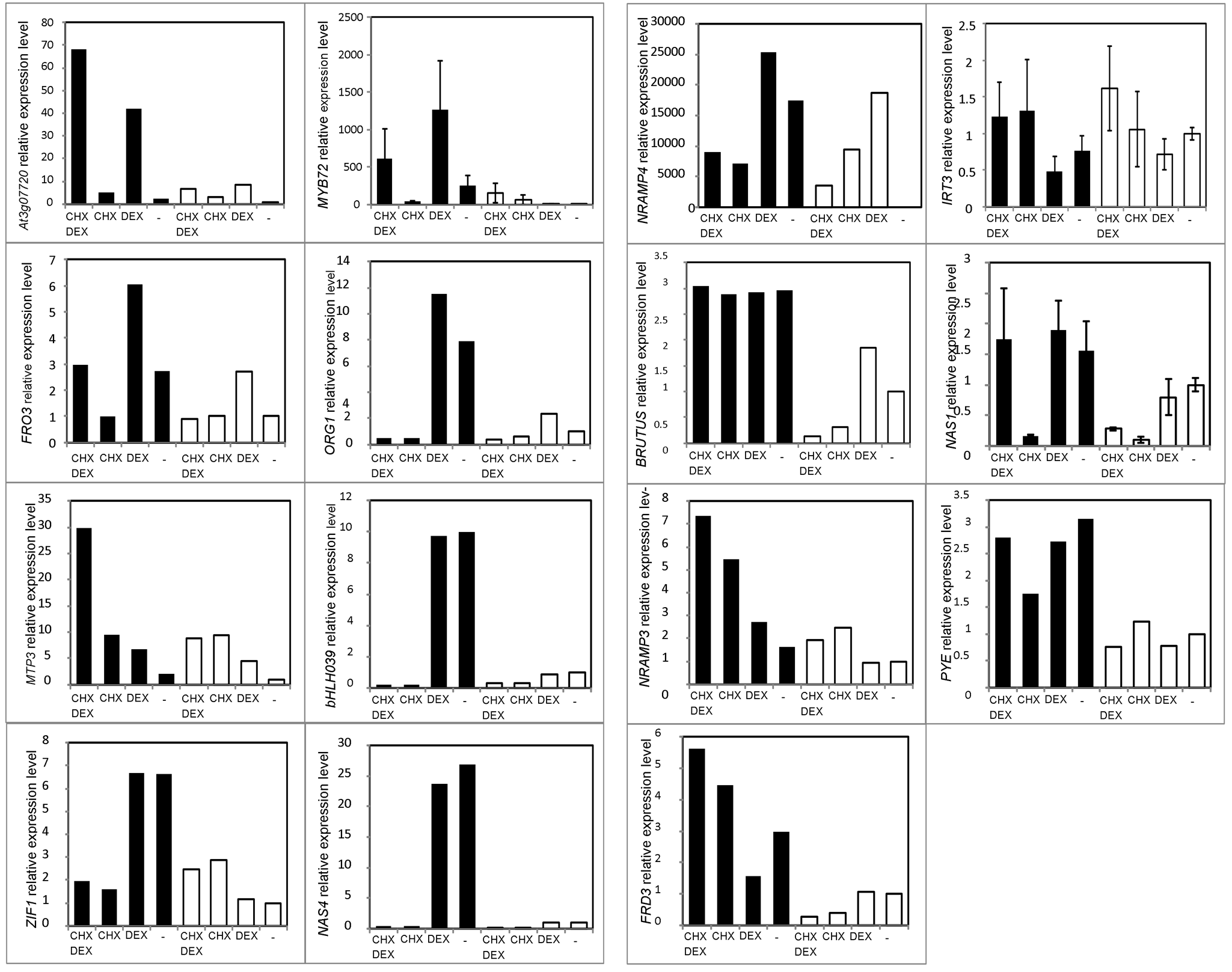

Supplement: Figure S1 — Expression of selected iron-responsive genes in fit-2/ 35S::FIT:GR plants. 8-day-old plants were grown on 1/2 MS without iron (black) or supplemented with 100 µM Fe (white). Roots were then transferred to liquid media with the same iron content but supplemented as follows: CHX DEX: 100 µM cycloheximide for one hour then 30 µM dexamethasone was added and incubated for 3 hours; CHX: 100 µM cycloheximide for one hour followed by a mock treatement for 3 hours, DEX: mock treated for one hour followed by 30 µM dexamethasone for 3 hours; or mock treatments only. Quantitative RT-PCR results are presented for with each gene’s relative expression level (REL) as indicated. When standard error bars are present n = 3–4. (TIF) [file pone.0044843.s001.tif]

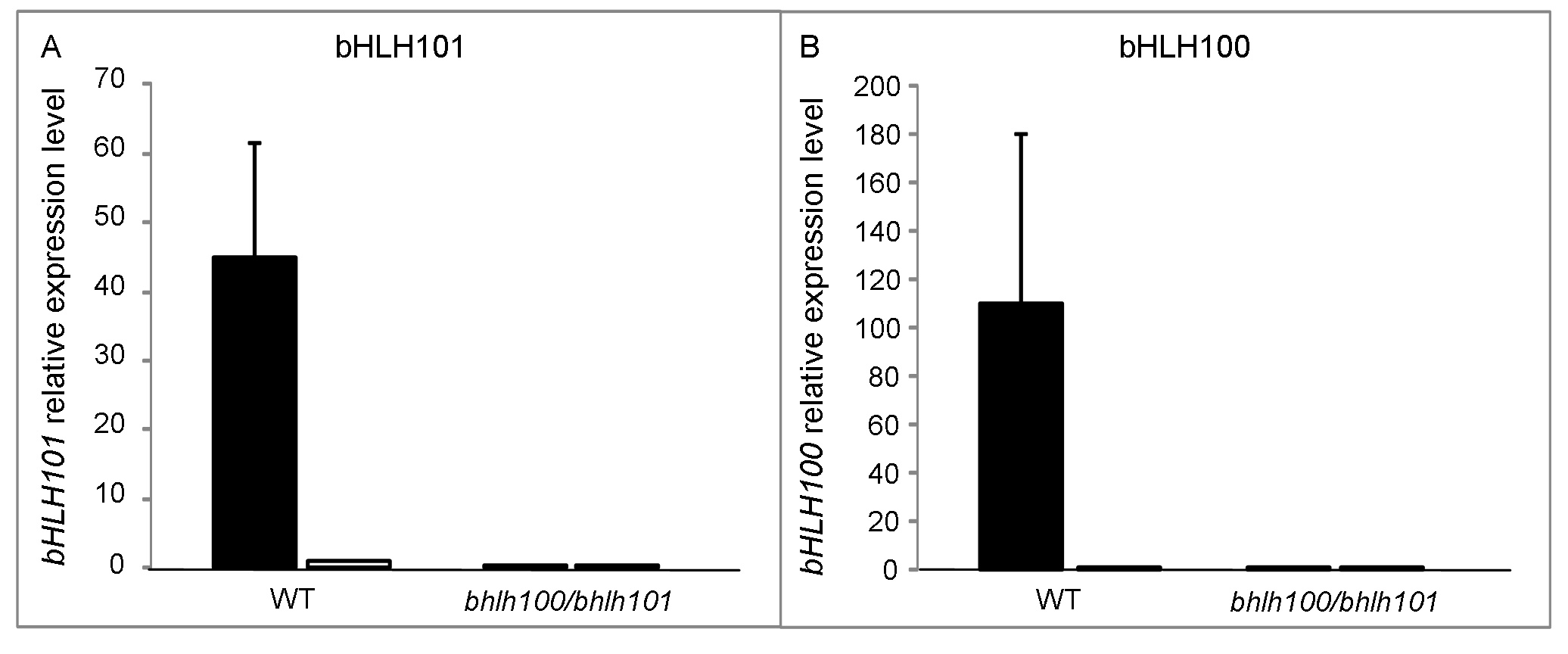

Supplement: Figure S2 — bhlh100/bhlh101 plants do not express bHLH100 or bHLH101. Quantitative RT-PCR using primers specific for bHLH101 (A) and bHLH100 (B) performed on total RNA extracted from wild-type (WT) and double mutant plants (n = 3). Plants were grown either in the absence of iron (black bars) or presence of iron (white bars; n = 3). (TIF) [file pone.0044843.s002.tif]

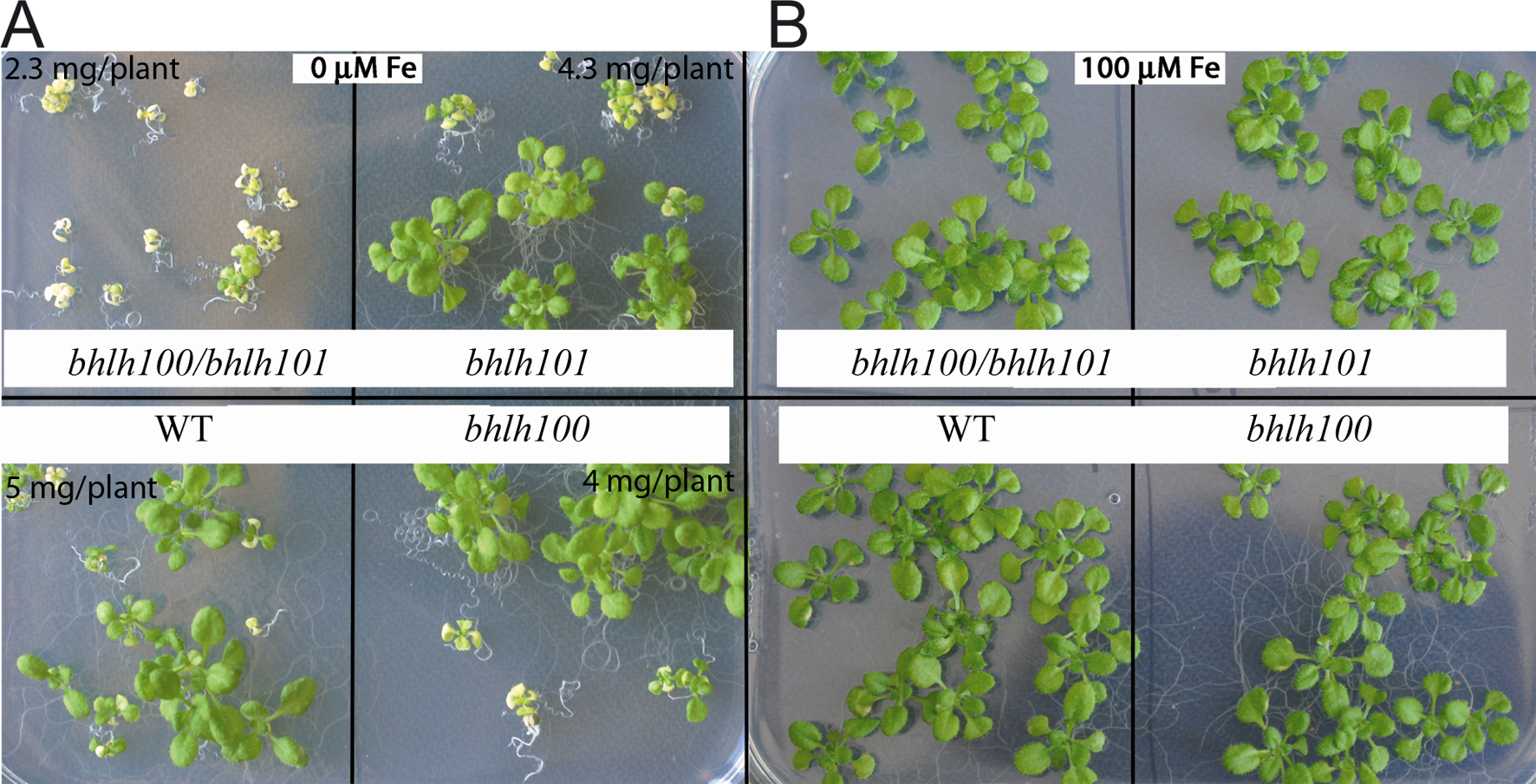

Supplement: Figure S3 — bhlh100 and bhlh101 single mutants do not display growth defects on low iron. 3-week-old wild-type (WT), bhlh100, bhlh101, and bhlh100/bhlh101 were grown on 1/2 MS plates without added iron (A) or with 100 µM Fe (B). The biomass of wild-type, bhlh100, bhlh101 and bhlh100/bhlh101 plants grow under iron-limited conditions (A) is shown. (TIF) [file pone.0044843.s003.tif]

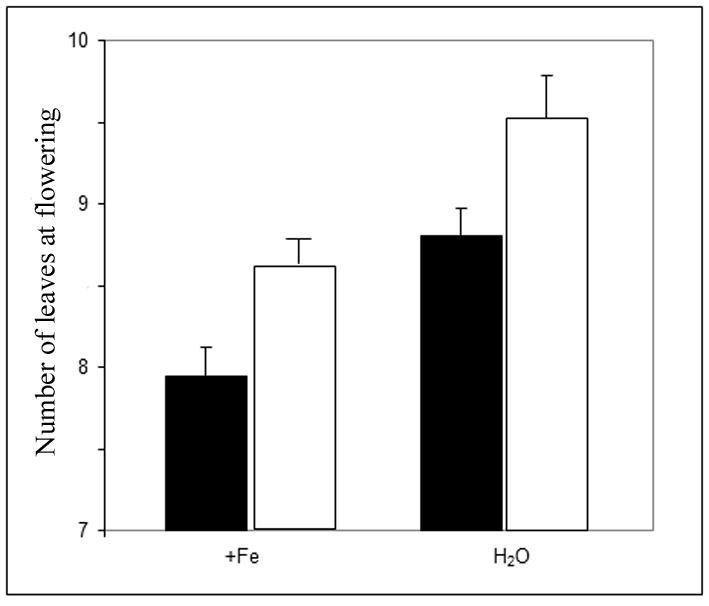

Supplement: Figure S4 — Flowering time of wild-type and bhlh100/bhlh101 plants supplemented with iron. bhlh100/bhlh101 plants flower late irrespective of iron nutrition. Plants were grown in the greenhouse and irrigated with either sequestrene (+Fe) or water from the start of cultivation. Leaves were counted at the time of flowering. For both watering treatments, wild-type plants (WT, black) had significantly less leaves at time of flowering than the double mutant (white) (p-values of 0.0098 and 0.0272 respectively). (TIF) [file pone.0044843.s004.tif]

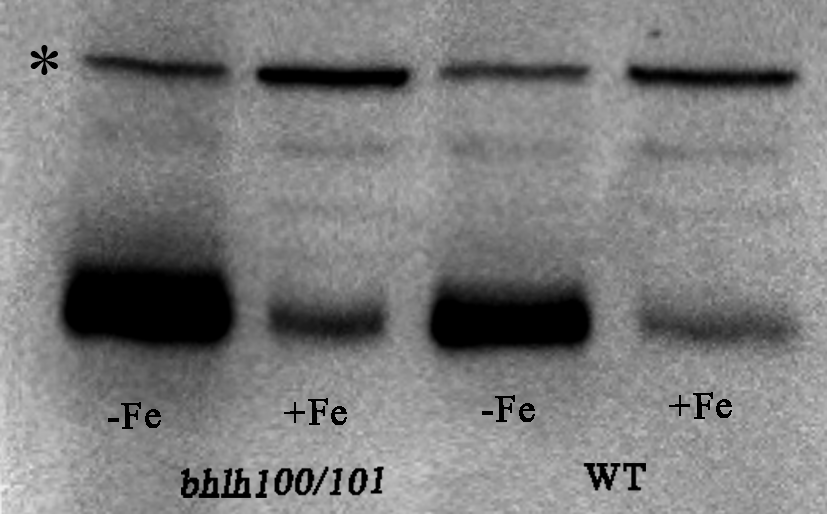

Supplement: Figure S5 — IRT1 protein accumulates to the same level in both wild-type and bhlh100/bhlh101 plants. Western blot of total protein extracts from 10-day-old wild-type (WT) and bhlh100/bhlh101 roots grown on 1/2 MS without added iron (–Fe) or with 100 µM Fe (+Fe). * indicates non-specific band is used as loading control. (TIF) [file pone.0044843.s005.tif]
